# Seven-Year Results for RESILIA Tissue in Bicuspid Aortic Valve Replacement Patients: Age and Valve Size Considerations

**DOI:** 10.1093/icvts/ivaf176

**Published:** 2025-08-01

**Authors:** Michael Salna, Joseph E Bavaria, David Heimansohn, Thomas Beaver, Bartley Griffith, Lars G Svensson, Philippe Pibarot, Michael A Borger, Vinod H Thourani, Eugene H Blackstone, Lorraine D Cornwell, John D Puskas, Hiroo Takayama

**Affiliations:** Division of Cardiac, Vascular and Thoracic Surgery, New York-Presbyterian/Columbia University Irving Medical Center, New York, NY, 10032, United States; Department of Cardiovascular Surgery, Jefferson Health, Philadelphia, PA, 19107, United States; St. Vincent Heart Center of Indiana, Indianapolis, IN, 46260, United States; Division of Cardiovascular Surgery, University of Florida Health, Gainesville, FL, 32611, United States; Department of Surgery, University of Maryland Medical Center, Baltimore, MD, 21201, United States; Department of Thoracic and Cardiovascular Surgery, Cleveland Clinic, Cleveland, OH, 44195, United States; Department of Cardiology, Québec Heart and Lung Institute, Laval University, Quebec, G1V 4G5, Canada; University Department of Cardiac Surgery, Heart Center Leipzig, Leipzig, 04289, Germany; Department of Cardiovascular Surgery, Piedmont Heart Institute, Marcus Valve Center, Atlanta, GA, 30309, United States; Department of Thoracic and Cardiovascular Surgery, Cleveland Clinic, Cleveland, OH, 44195, United States; Division of Cardiothoracic Surgery, Baylor College of Medicine, Houston, TX, 77030, United States; Division of Cardiothoracic Surgery, Emory University School of Medicine, Atlanta, GA, 30342, United States; Division of Cardiac, Vascular and Thoracic Surgery, New York-Presbyterian/Columbia University Irving Medical Center, New York, NY, 10032, United States

**Keywords:** bicuspid aortic valve, aortic stenosis, aortic regurgitation, aortic valve replacement, RESILIA, clinical outcomes

## Abstract

**Objectives:**

Patients with bicuspid aortic valve disease requiring surgical aortic valve replacement are often younger and want to avoid lifelong anticoagulation. A multicentre single-arm non-randomized study, the COMMENCE trial, studied outcomes of RESILIA tissue aortic valves in bicuspid aortic valve patients through 7 years of follow-up.

**Methods:**

Of 672 patients who underwent surgical replacement of native aortic valves, 214 had bicuspid and 458 had tricuspid aortic valves. Propensity score analyses with inverse probability of treatment weighting were utilized to minimize bias due to measured confounders. Linear mixed-effect models compared longitudinal changes in haemodynamic parameters.

**Results:**

Patients with bicuspid were significantly younger than those with tricuspid aortic valves—mean age of bicuspid: 59.8 (12.4) vs tricuspid: 70.2 (9.5) years; *P* < .001; 39/214 (18%) bicuspid aortic valve patients were <50 years old. There was no evidence of structural valve deterioration in any bicuspid aortic valve patients over 7 years of follow-up. At 7 years, there was no significant difference between bicuspid and tricuspid aortic valve patients in propensity score- and age-adjusted survival (91.9% vs 88.1%, respectively; *P* = .35), stroke, or reoperation. Among bicuspid aortic valve patients <65 years of age, there was no significant difference in prosthetic valve effective orifice areas and mean gradients between 3 months and 7 years postoperatively.

**Conclusions:**

Patients with bicuspid aortic valves had excellent outcomes with RESILIA tissue valves at 7 years with no evidence of structural valve deterioration. These results suggest a durable alternative for carefully selected younger patients wishing to avoid anticoagulation.

**Clinical Trial Registration Number:**

NCT01757665.

## Graphical abstract

**Figure:**
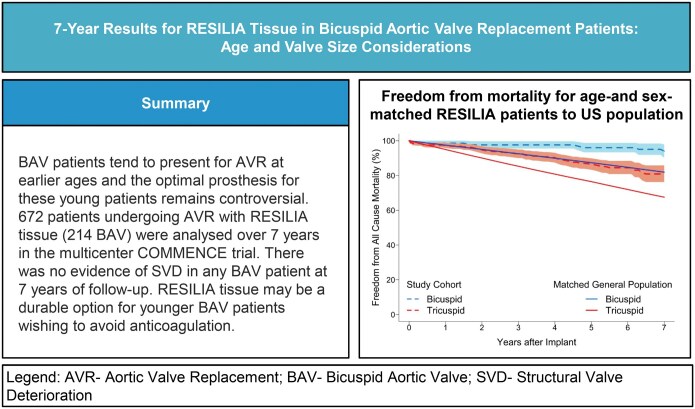

## INTRODUCTION

Currently, there are approximately 7 million people with bicuspid aortic valves (BAV) living in the United States, comprising ∼2% of the population. It is estimated that the cumulative lifetime risk of developing at least moderate aortic stenosis (AS) or aortic regurgitation (AR) exceeds 80% in BAV patients.^1^ Unlike patients with tricuspid aortic valves (TAV), BAV patients tend to present with severe AS and AR earlier—in their 50s or 60s rather than in their 70s or 80s.^2^ Because studies have shown that aortic valve replacement (AVR) normalizes life expectancy in BAV patients with severe AS,^3^ it is not surprising that these patients make up nearly 50% of patients seeking AVR.

Recently, surgical AVR (SAVR) with tissue valves has been occurring at increasing numbers in younger patients, driven by the potential for future transcatheter intervention and avoidance of anticoagulation with this treatment.^4^ However, it is well-known that young age is one of the most significant risk factors for early structural valve deterioration (SVD) in bioprosthetic valves.^5–7^ Thus, together with the recent guidelines and lowering age cutoff for bioprosthetic SAVR,^8^ the creation of a reliable and durable biologic aortic valve is of critical importance.

This unmet need drove the development of RESILIA tissue—bovine pericardium with novel integrity-preservation technology preventing calcification and permitting dry storage. The COMMENCE trial, originally published in 2017, reported early outcomes of this novel RESILIA tissue in SAVR.^9^ To date, the longest term follow-up from this trial has been 7 years, with encouraging results for SAVR bioprostheses.^10^ At present, literature is lacking for SAVR in the relatively young BAV patient population. Previously, a sub-analysis of the COMMENCE trial reported outcomes of BAV patients through 5 years of follow-up with no SVD reported.^11^^,^^12^ In the present study, follow-up through 7 years is reported for the COMMENCE trial with a focus on young BAV patients.

## METHODS

### Study design and patients

The COMMENCE trial is a multicentre, single-arm, prospective, observational US Food and Drug Administration Investigational Device Exemption (FDA IDE) study designed to test the safety and efficacy of bioprosthetic aortic valves incorporating RESILIA tissue. The details of this study including eligibility have been described.^11^ In brief, adult patients requiring AVR with or without concomitant procedures were enrolled. Those undergoing emergency AVR, having endocarditis within 3 months, hypertrophic obstructive cardiomyopathy, or undergoing concomitant repair/replacement of another valve were excluded. The definition of SVD was based on Akins et al, as dysfunction or deterioration involving the operated valve (excluding infection or thrombosis), as determined by reoperation, autopsy, or clinical investigation.^12^ The FDA determined this definition would be used for the COMMENCE study at the time of trial initiation in 2012. Although SVD and NSVD are known causes of bioprosthetic valve failure, this was not specifically assessed or adjudicated in the present study. From January 2013 to March 2016, 672 patients underwent native AVR, 214 had bicuspid aortic valves and 458 had tricuspid valves.

### Statistical methods

Continuous variables were summarized by mean and standard deviation, and categorical variables were summarized by counts and percentages. The Kaplan-Meier method with log-rank test was used to compare freedom from each safety event between the BAV and TAV groups.

To account for imbalances in patient characteristics between the 2 study groups, we applied propensity score (PS) analyses with inverse probability of treatment weighting (PS-IPTW) to minimize bias due to measured confounders.^13^ The PS for each patient was calculated based on *a priori* selected clinically relevant variables including age, sex, BSA, New York Heart Association (NYHA) class, concomitant surgery, endocarditis, congestive heart failure, renal failure/insufficiency, previous cardiac surgery, coronary artery disease, peripheral artery/vascular disease, chronic obstructive pulmonary disease, diabetes, pulmonary hypertension, liver disease, and valve size. Left ventricular ejection fraction was not included due to incomplete data (*n* = 155, 23%). Inverse probability weight was calculated for each patient based on the estimated PS. We evaluated covariate balance using absolute standardized mean differences (**Figure S1**).^4^ Adjusted 7-year freedom from each safety event probability was estimated and compared based on double adjustment combining PS-IPTW and Cox regression adjusted for age to remove residual confounding bias (ie, absolute standardized mean difference for age was greater than 0.20 after PS-IPTW).^14^^,^^15^ In addition, overall survival of our study cohort was compared with expected survival of the age- and sex-matched US general population using function *survexp* in statistical R package *survival*. Mixed-effects models, adjusted for the logit of PSs and age, were performed to evaluate haemodynamic trends.

To quantify patient-prosthesis mismatch (PPM) at 3 months, PPM was defined as Severe (indexed effective orifice area [EOAi] ≤0.65 if BMI < 30 or EOAi ≤ 0.55 if BMI ≥ 30), Moderate (EOAi > 0.65 & ≤0.85 if BMI < 30 or EOAi > 0.55 & ≤0.70 if BMI ≥ 30), or None/Mild (EOAi > 0.85 if BMI < 30 or EOAi > 0.70 if BMI ≥ 30).^16^ We further studied severe PPM patients with elevated mean gradient (>20) and valve dysfunction (Doppler velocity index [DVI] <0.25).

All tests were 2-sided, and statistical significance was set at *P* < .05. Analyses were performed using *R* version 4.2.3 (R Core Team, 2023).

## RESULTS

### Patients and characteristics

A total of 672 patients were successfully implanted with the study valve between January 2013 and March 2016. In this cohort, 214 patients had BAV, 105 of which consented to extended follow-up, and 90 completed the 7-year follow-up; 458 patients had TAV, 114 of which consented to extended follow-up, and 100 completed the 7-year follow-up. After completing their 5-year follow-up visit, 7 BAV patients consented to participate in extended follow-up, but could not complete their 7-year visit for various reasons: 2 died of cancer, 1 valve explant due to enlarging aneurysm (non-valve-related), and 7-year visit completion was pending for 4 patients at the time of data lock.

Baseline characteristics were previously reported.^9^ The most common indications for AVR were stenosis (51%), stenosis with insufficiency (39%), and pure insufficiency (7%).

Patients with BAV were significantly younger in age—mean age 59.8(12.4) vs TAV: 70.2(9.5) years; *P* < .001; 39/214 (18%) BAV patients were less than 50 years of age. Mean Society of Thoracic Surgeons PROM scores were 1.2 (1.0) for BAV and 2.3 (2.0) for TAV patients, and EuroScore II scores were 1.0 (1.7) for BAV and 2.7 (2.9) for TAV patients.

Valves in BAV patients also tended to be larger. The most common valve sizes implanted were 25 mm in BAV (33%) and 23 mm in TAV (35%) patients; 63% of BAV patients received a size 25 mm or greater, in contrast to 40% of TAV patients (*P* < .001).^11^

### Clinical outcomes

At 7 years, BAV and TAV patients were comparable across all measured postoperative outcomes.^9^ There was no significant difference between BAV and TAV patients in PS-IPTW and age-adjusted survival (91.9% BAV vs 88.1% TAV; *P* = .35), stroke, or reoperation (**Table 1**). In the BAV cohort, there were 2 events of valve thrombosis. Four BAV patients required repeat valve replacement (3 from endocarditis and another during reoperation for an ascending aortic aneurysm without any valve dysfunction). No BAV patients had evidence of SVD or paravalvular leak (PVL). In the TAV cohort, there was no valve thrombosis. Five TAV patients required repeat AVR (4 due to endocarditis and 1 due to SVD from calcification 5.3 years post-operatively). Two TAV patients underwent reinterventions (1 due to SVD—restricted leaflet motion of uncertain aetiology and 1 due to major PVL), and 1 TAV patient required surgery (non-explant) for major PVL.

**Table 1.** PS-IPTW and Age-Adjusted Freedom from Events at 7 Years (%) for All Patients

Event	BAV cohort (N = 213)	TAV cohort (N = 451)	*P*-value^a^
Mortality	91.9 (88.2, 95.8)	88.1 (82.0, 94.6)	.35
Valve-related mortality	98.1 (96.4, 99.8)	96.1 (93.9, 98.4)	.23
Reoperation	98.3 (96.9, 99.8)	97.0 (94.7, 99.3)	.36
Study valve explant	98.5 (97.3, 99.8)	98.3 (96.6, 100.0)	.84
Stroke	92.6 (89.5, 95.8)	95.1 (92.6, 97.7)	.28
Valve thrombosis^b^	NA	NA	NA
Endocarditis	98.9 (97.6, 100.0)	96.1 (93.7, 98.4)	.07
Structural valve deterioration^c^	NA	NA	NA
Major PVL^d^	NA	NA	NA
PPI	92.1 (88.9, 95.4)	91.9 (88.4, 95.5)	.94
Valve-related PPI	98.9 (98.0, 99.9)	99.8 (99.4, 100.0)	.16

Abbreviations: PPI, permanent pacemaker implantation; PVL, paravalvular leak.

a Doubly robust estimation combining IPTW and Cox regression adjusted for age.

b NA for analyses due to too small event numbers (ie, 2 events in BAV and 0 events in TAV).

c NA for analyses due to too small event numbers (ie, 0 events in BAV and 2 events in TAV).

d NA for analyses due to too small event numbers (ie, 0 events in BAV and 3 events in TAV).

**Figure 1.**
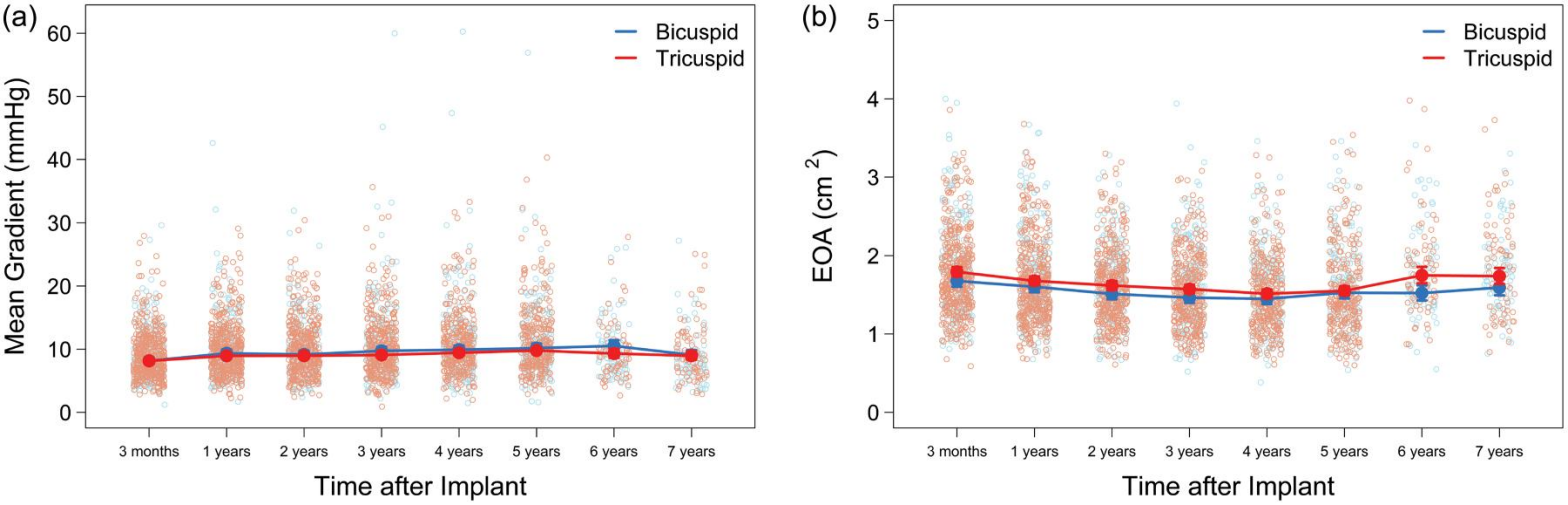
Adjusted Echo-Derived (A) Mean Gradients (mmHg) and (B) Effective Orifice Area (cm^2^)

Mean gradients and EOAs were clinically stable over the study period (**Figure 1**). Two BAV patients developed elevated gradients over the study period: (1) A 26-year-old woman received a 19 mm prosthesis with a mean gradient of 17.8 mmHg observed 3 months postoperatively. Nearly 2 years after the procedure, the patient’s mean gradient rose to 31.9 mmHg during her first pregnancy. She underwent a C-section for her second pregnancy at 4.4 years and was asymptomatic during her last follow-up at 5 years with a gradient of 40.3 mmHg. (2) A 67-year-old woman received a 19 mm study valve and a pacemaker. At her 3-month postoperative visit, the mean gradient was 29.6 mmHg, which gradually increased to 47.4 mmHg at her 4-year follow-up. Notably, she maintained NYHA Class I symptoms throughout the study period, had no thrombosis, and no NSVD reported; she consequently did not undergo any intervention.

Of the 617 subjects who had available EOAi, mean gradient, and DVI data at 3 months, none had evidence of severe PPM with mean gradient >20 mmHg and DVI < 0.25.

### Comparison to US population

**Figure 2.**
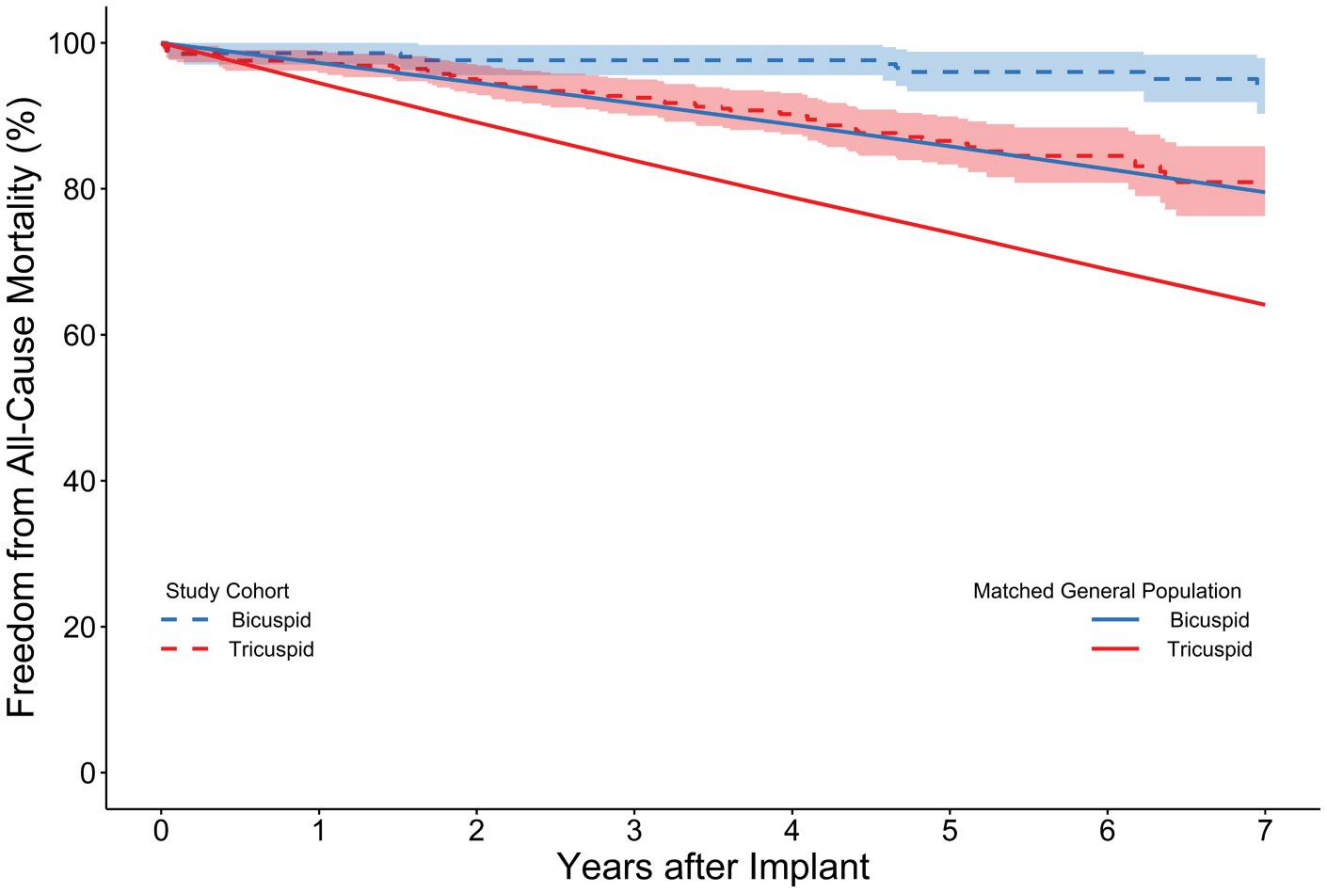
Freedom from All-Cause Mortality for Age- and Sex-Matched to US Population

In an additional age- and sex-matched analysis, COMMENCE patient data were compared with US census data.^17^ Survival was not reduced in BAV and TAV patients relative to their respective age- and sex-matched cohorts (**Figure 2**). There was also no significant difference in survival between either BAV or TAV patients who underwent AVR and those matched patients who did not. However, it is important to recognize the inherent differences in health status between these groups.

### Outcomes among young patients

In COMMENCE patients ≤65 years of age, there were no significant differences in all clinical outcomes (**Table 2**). Furthermore, within this young cohort, there were no significant differences in individual BAV patient EOAs or mean gradients between 3 months and 7 years postoperatively. Mean gradient at 3 months was 9.39 (4.27) mmHg and at 7 years: 9.13(4.47) mmHg (**Figure S3**).

**Table 2.** PS-IPTW and Age-Adjusted Freedom from Events at 7 Years (%) for Patients ≤65 Years

Freedom from event	BAV cohort (N = 133)	TAV cohort (N = 108)	***P*-value** ^a^
Mortality	99.2 (98.0, 100)	96.7 (93.1, 100)	.20
Valve-related mortality	99.4 (98.7, 100)	99.1 (97.2, 100)	.68
Reoperation	95.4 (91.4, 99.5)	97.8 (93.9, 100)	.50
Study valve explant	95.4 (91.4, 99.5)	97.8 (93.9, 100)	.50
Stroke	96.9 (93.9, 100)	97.4 (95.1, 99.8)	.81
Endocarditis	96.2 (92.2, 100)	94.9 (88.9, 100)	.75
PPI	94.3 (90.6, 98.1)	94.1 (89.5, 99.1)	.97
Valve-related PPI^b^	NA	NA	NA

Abbreviation: PPI, permanent pacemaker implantation.

a Doubly robust estimation combining IPTW and Cox regression adjusted for age.

b NA for analyses due to too small event numbers (ie, 2 events in BAV and 1 event in TAV).

**Figure 3.**
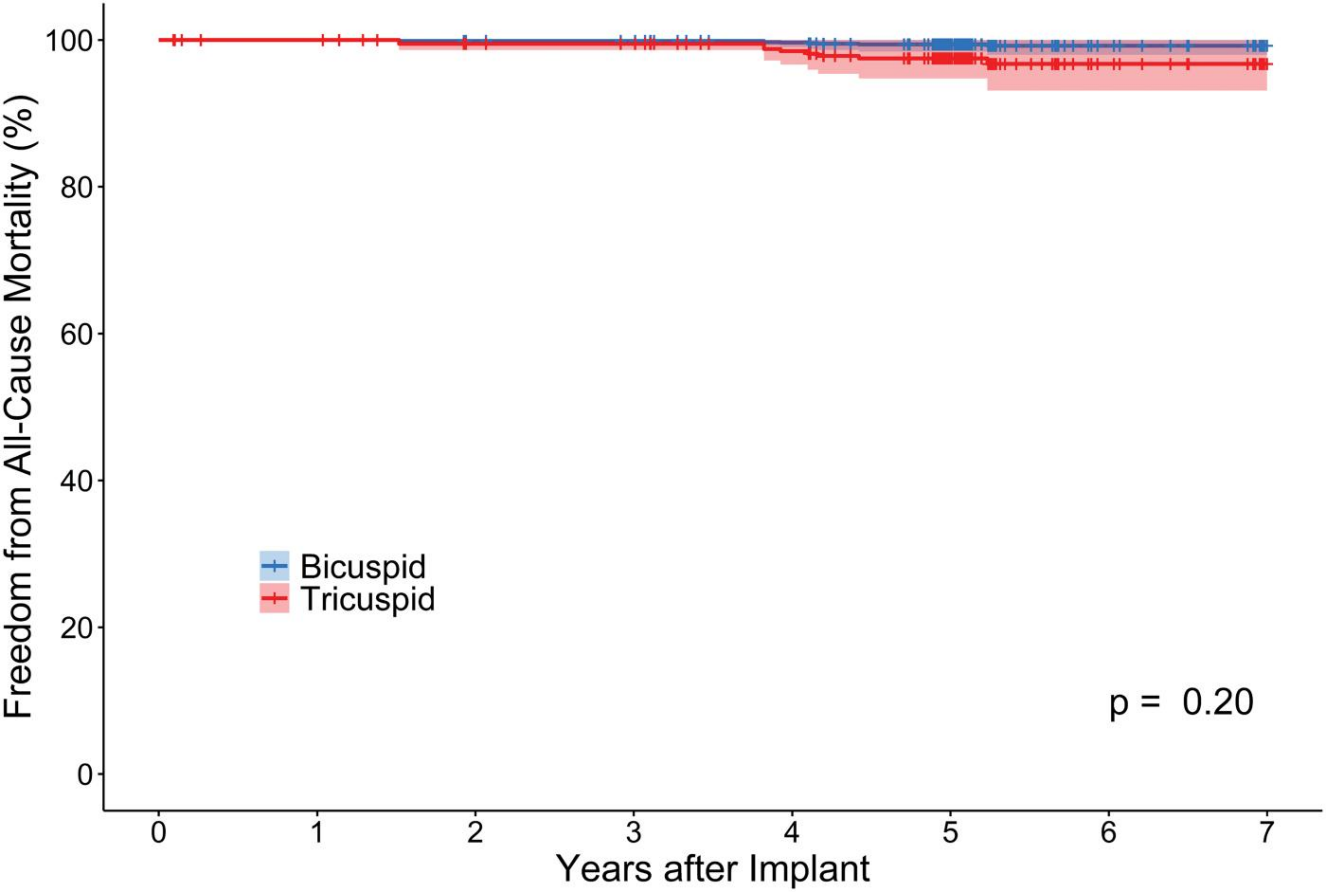
PS-IPTW and Cox Regression Adjusted Freedom from All-Cause Mortality for Young (Age ≤65 years) Patients

There were 4 BAV patients ≤65 years old who received 19 mm valves and 18 who received 21 mm valves. At 3 months, median mean gradients were 15.2 mmHg (IQR [interquartile range]: 12.5-20.9) and 12.9 mmHg (IQR: 12.9-18.1) for 19 mm and 21 mm valves, respectively. At 5 years, median mean gradients had increased to 32 mmHg (*n* = 3; IQR: 25.8-44.5) and 16.2 (*n* = 15; IQR: 13.0-21.1) for 19 mm and 21 mm valves, respectively. These 4 BAV patients did not pursue extended follow-up. Lastly, propensity-matched BAV and TAV patients within this cohort demonstrated no difference in survival by 7 years (Graphical abstract, **Figure 3**, **Figure S2**).

### Aortic root dimensions

**Figure 4.**
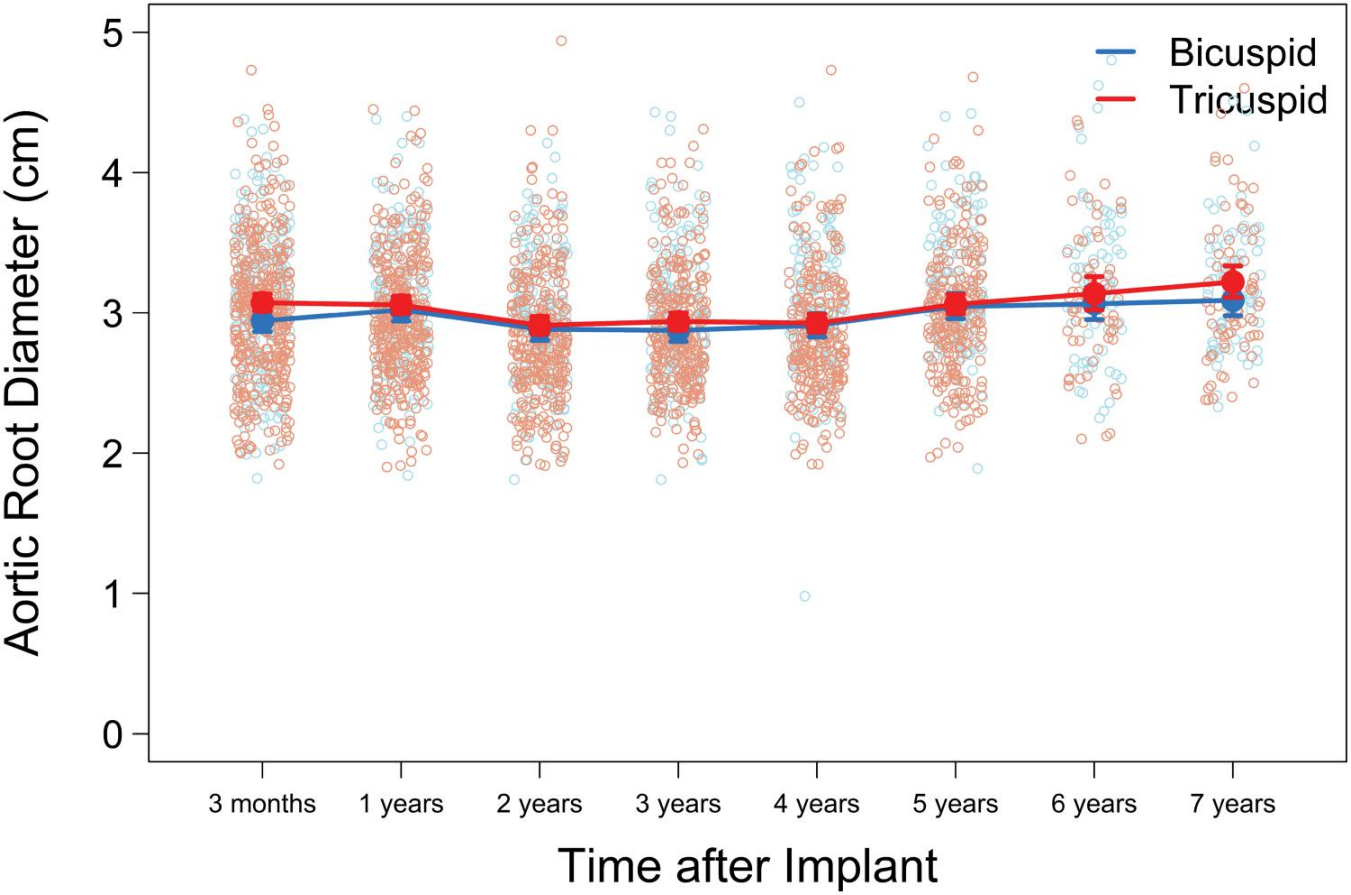
Mixed-Effect Models of Change in Aortic Root Diameter in BAV and TAV Cohorts, Adjusting by Age, BSA, and Valve Size

There were no significant differences in echocardiographic-derived aortic root diameters between BAV and TAV patients across the 7 years of follow-up. Moreover, linear mixed-effects model adjusted for age, BSA, and valve size (**Figure 4**) demonstrated mean root diameter increased slightly over the study period, from 3 months (BAV: 2.94 cm, TAV: 3.07 cm; *P* = .01) to 7 years (BAV: 3.09 cm, TAV: 3.22 cm; *P* = .11). These modest differences remained for patients <65 years.

## DISCUSSION

This study was a 7-year sub-analysis of the COMMENCE trial, a US FDA IDE trial adjudicated by a clinical events committee and echo core lab that evaluated the efficacy of RESILIA tissue aortic valve prostheses in BAV patients. This study demonstrated 3 key findings in BAV patients at 7 years: first, RESILIA tissue demonstrated safety and durability, as demonstrated by no evidence of SVD or PVL. Second, echocardiographic data of EOAs and transvalvular gradients were promising, even in a subgroup of young patients (≤65 years old). Finally, BAV patients who underwent SAVR with RESILIA tissue valves showed life expectancy curves mirroring those of age- and sex-matched individuals from the general US population, suggesting a normalization of life expectancy. Additionally, this study is unique as we utilized PS-IPTW methodology to balance baseline characteristics and evaluated change in aortic root diameter over 7 years. The results of the COMMENCE trial suggest that SAVR is an excellent option in this young, low-risk BAV population. This trial is ongoing, with patients followed through 10-years of follow-up.

Historically, a principal disadvantage of bioprosthetic valves in young patients was durability, which lead to high rates of SVD and likelihood of reoperation. Johnston et al reported 20-year follow-up on 12 569 patients who received the Carpentier-Edwards Perimount aortic valve and demonstrated good durability with only 15% of valves requiring explant for SVD.^6^ However, subgroup analysis of patients younger than 60 years old had a 5.6% rate of explant for SVD by 10 years. Taken together with the general incidence of explant for SVD, the true incidence of SVD may be underestimated, with some studies reporting 25%-35% of bioprosthetic aortic valve patients presenting with variable degrees of SVD in less than 10 years.^5^^,^^7^^,^^18^ Consequently, SVD would have been anticipated to be higher in our BAV cohort, which was almost 10.5 years younger than TAV patients. This was not the case. Even when adjusting for age, valve size, and BSA, there remained no difference in SVD results between BAV and TAV subgroups with excellent haemodynamic follow-up. Bartus et al compared 5-year SVD rates of RESILIA tissue valves to those from the PARTNER 2 A contemporary AVR arm and found, in propensity matched cohorts, a 1.0% vs 4.8% rate, respectively (*P* = .03).^12^ There was no evidence of SVD in any BAV patients at 7 years as defined by the joint STS/AATS/EACTS Guidelines,^19^ even among patients ≤65 years old. However, there were instances of restricted leaflets and high mean gradients in small prosthesis BAV patients. Patient BAV aetiology did not appear to be an independent risk factor for earlier SVD in the trial, nor did age for RESILIA tissue valves at 7 years.

Overall, young BAV patients had excellent haemodynamic results 7 years after SAVR. Transvalvular gradients, EOAs, and PPM have direct implications for bioprosthetic valve durability and patient outcomes.^20^ In the COMMENCE trial, BAV patients maintained stable low mean gradients and high EOAs throughout the 7-year study period. Among those BAV patients ≤65 years of age, there were no significant differences in these parameters between the 3-month and 7-year time points, suggesting sustained durability of the RESILIA tissue valves. Median mean gradients did increase considerably to 5 years in 4 BAV patients who received a 19 mm valve. These patients did not pursue extended follow-up, but due to limited sample size (*n* = 4), it is not possible to conclude how RESILIA tissue performs in these patients.

One particularly challenging cohort is that of young BAV patients with a small aortic root. A total of 34 (16%) of BAV patients received a size 19 mm or 21 mm valve, though notably the mean BSA of this sub-group was only 1.78 m^2^. While only 1.9% of the entire COMMENCE study population underwent root enlargement, these techniques have become more widely adopted in clinical practice today.

Aortic growth rate is known to be significantly higher in patients with BAV. However, among BAV patients with normal or mildly enlarged diameters at the time of AVR alone, late aortic events are rare. Svensson et al studied 1449 BAV patients who underwent AVR with aortic diameters <4.5 cm at the time of surgery and found that only 3 of them had late aortic events over 11 years.^21^ While the present trial lacked computed tomography imaging data to assess aortic size, echocardiographic data supported this finding with minimal change in aortic root diameters among BAV patients over 7 years of follow-up.

As younger patients more frequently request tissue valves, valve technology is evolving to become safer and more durable to adapt to this growing demand. Historically, mechanical valves were recommended for young patients needing AVR. Today, over 85% of surgical AVRs are bioprosthetic, and younger patients are increasingly requesting tissue valves to avoid anticoagulation.^22^ However, guidelines and data remain in conflict. Recent Swedish data demonstrated survival advantages of mechanical valves in 50- to 69-year-old patients^23^ and the 2020 American Heart Association/American College of Cardiology guidelines recommend mechanical aortic valves for patients <65 years of age. The 2021 European Society for Cardiology/European Association for Cardio-Thoracic Surgery guidelines also recommend mechanical aortic valves for patients <60 years.^24^ In contrast, California registry data by Goldstone et al concluded that the survival benefits attributed to mechanical valves in young patients end at age 55.^25^ These results echoed a 2019 retrospective multicentre trial of 9388 AVR patients where there was no difference in adjusted long-term survival between mechanical and tissue prostheses at 15 years, though not unexpectedly a higher rate of reoperation among tissue valve patients.^4^ Our results suggest that AVR with a RESILIA tissue valve normalized the survival curves of BAV patients to age- and sex-matched controls who did not undergo AVR. Among patients ≤65 years of age, there were no adverse events or evidence of SVD. These findings were echoed by the recently published 1-year follow-up data of the INDURE trial, which found no evidence of SVD in 421 patients (73% BAV) under age 60 undergoing AVR with the INSPIRIS valve.^26^ In their totality, these results suggest that RESILIA tissue valves represent a reliable option for minimizing risks to younger patients.

### Limitations

This sub-analysis is subject to the limitations and biases inherent in any retrospective analysis of prospective data. Computed tomography was not a requirement in the protocol design for the COMMENCE trial, and thus, aortic and annular dimensions were not proactively collected and nor were bicuspid classifications. Data on left ventricular ejection fraction were not available for many patients, owing to many of the trial sites using visual estimation to quantify ejection fraction rather than the Echo Core Lab’s standard of Simpson’s volumetric method. As previously reported, the COMMENCE trial did not include patients randomized to other valve types, thereby limiting comparisons to other tissue or mechanical valves. Patients in this study received valves from high volume surgical centres that utilized larger valve sizes so these results may not be generalizable to all surgical centres. There may be selection bias at the time that patients from the original trial cohort reconsented for extended follow-up, as reflected by a decrease in enrolled patients. Due to limited data in patients (*n* = 4) who received 19 mm valves, it is not possible to conclude how RESILIA performs in these patients; warranting future studies with smaller RESILIA tissue valves in BAV patients.

## CONCLUSION

In conclusion, this FDA IDE study continues to demonstrate that preoperative valve morphology—whether BAV or TAV—has little influence on SAVR outcomes. In the emerging era of lifetime aortic valve management, SAVR will continue to play an essential role, particularly in younger low-risk patients. At present, the results of COMMENCE at 7 years illustrate RESILIA tissue’s midterm durability, making it an excellent option for young patients and patients with bicuspid aortic valves wanting to avoid the complications associated with mechanical prostheses.

## Supplementary Material

ivaf176_Supplementary_Data

## Data Availability

All relevant data are included in the manuscript and its supporting files.
