# Supplementary figures and images for: Seven-Year Results for RESILIA Tissue in Bicuspid Aortic Valve Replacement Patients: Age and Valve Size Considerations
Source: Interdiscip Cardiovasc Thorac Surg. 2025 Aug 1;40(8):ivaf176. doi: 10.1093/icvts/ivaf176 (PMC12342796; doi:10.1093/icvts/ivaf176)

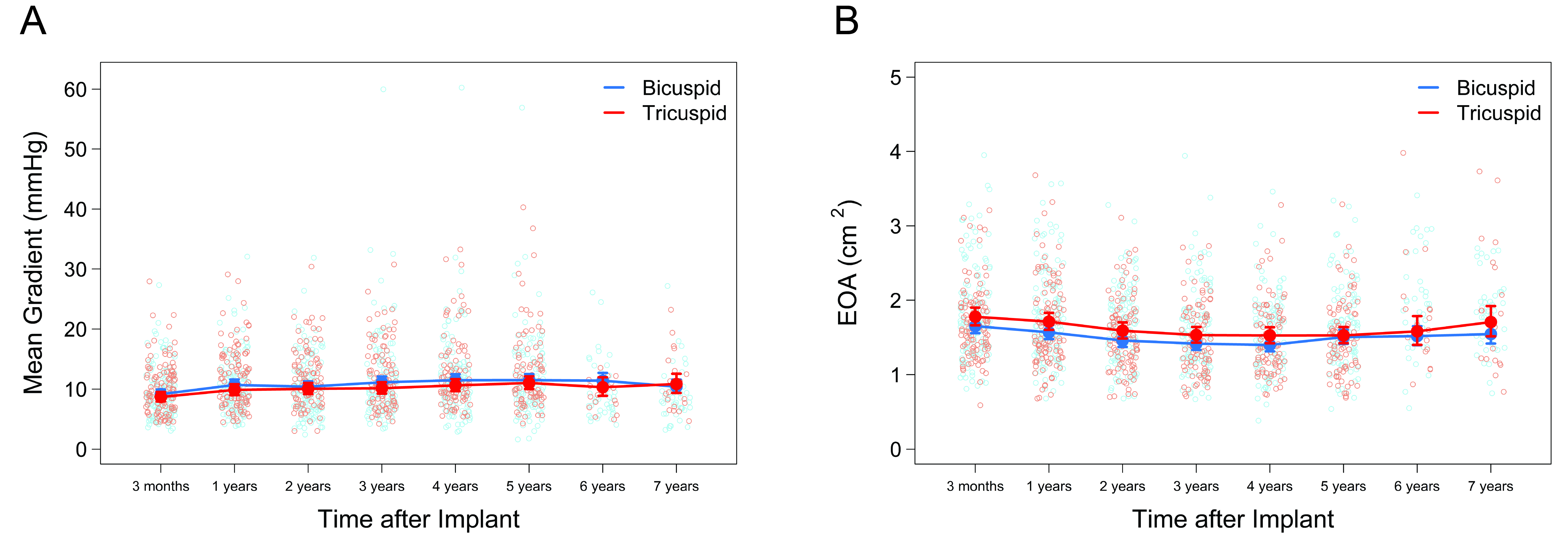

Supplement: ivaf176_Supplementary_Data [file ivaf176_supplementary_data.zip › Supp Figure 3.tif.tif]

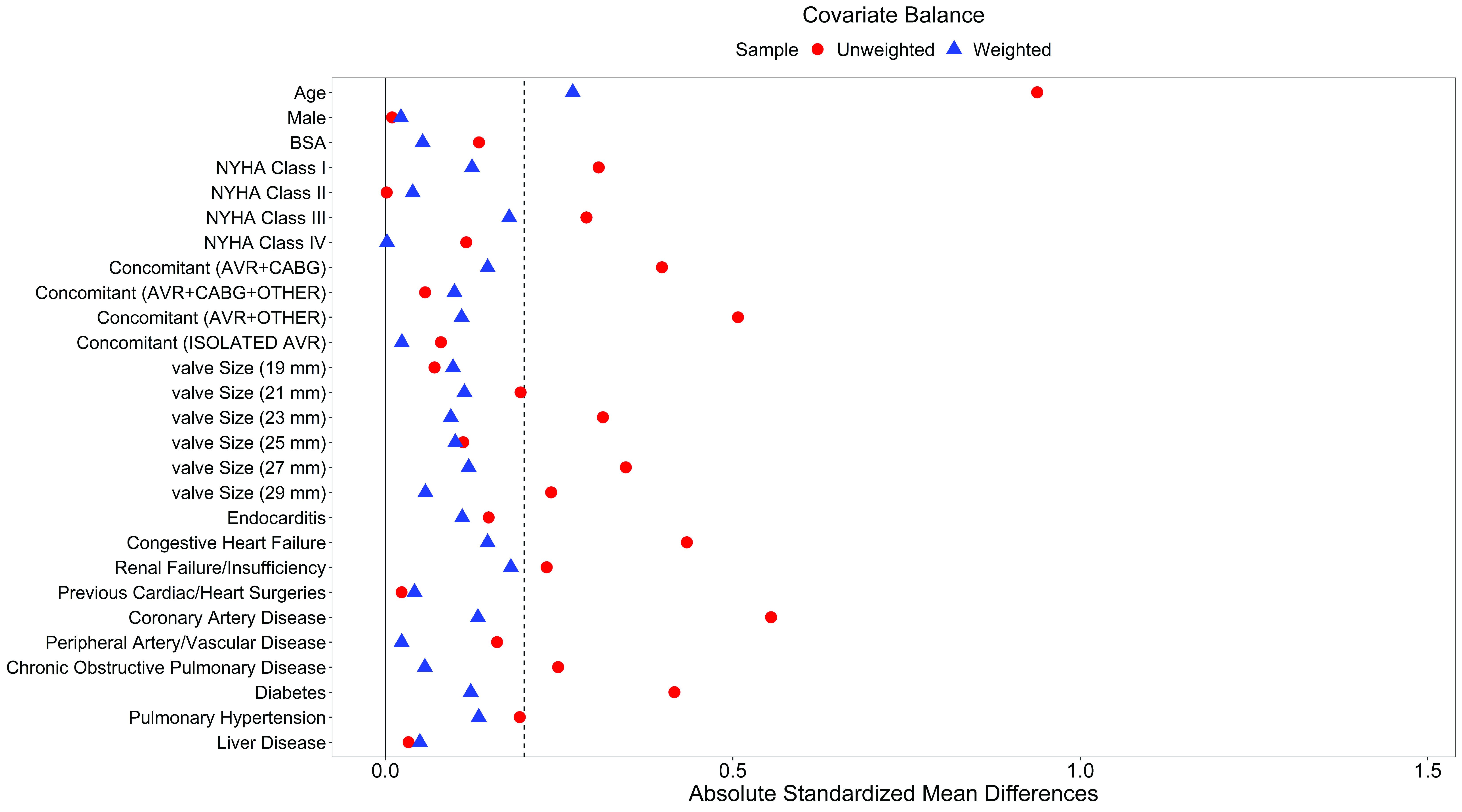

Supplement: ivaf176_Supplementary_Data [file ivaf176_supplementary_data.zip › Supp Figure 1.tif.tif]
